# Late Survival and Long‐Term Follow‐Up After Radical Resection of Advanced Renal Cell Carcinoma With Associated Venous Tumor Thrombus

**DOI:** 10.1002/jso.28020

**Published:** 2024-11-26

**Authors:** Calvin L. Chao, Nidhi K. Reddy, Maxime Visa, Shilajit D. Kundu, Mark K. Eskandari

**Affiliations:** ^1^ Department of Surgery, Division of Vascular Surgery Northwestern University Feinberg School of Medicine Chicago Illinois USA; ^2^ Department of Urology, Division of Urologic Oncology Northwestern University Feinberg School of Medicine Chicago Illinois USA

**Keywords:** inferior vena cava, Neves classification, renal cell carcinoma, venous reconstruction, venous tumor thrombus

## Abstract

**Background and Objectives:**

This study evaluates the prognostic value of venous tumor thrombus (VTT) in patients with advanced renal cell carcinoma (RCC) undergoing radical resection and inferior vena cava (IVC) thrombectomy.

**Methods:**

Retrospective review of patients with radical nephrectomy for RCC and associated VTT (2000−2024). Patients were dichotomized into Neves 0−II (infrahepatic) and Neves III−IV groups (suprahepatic) IVC involvement for univariate analysis.

**Results:**

A total of 64 patients (34 Neves 0−II and 30 Neves III−IV) were analyzed. No significant differences in patient or cancer characteristics. Neves III−IV was associated with greater blood loss (> 2 L) (62.1% vs. 37.9%, *p* = 0.02), greater intensive care unit length of stay (LOS) (4.4 vs. 1.4 days, *p* = 0.02), and postoperative LOS (11.0 vs. 6.5 days, *p* = 0.005). Overall, 30‐day mortality was only 1.6% with a mean follow‐up of 56.1 months. Local recurrence was 7.8% and IVC patency 96.9%. One‐year survival was 82.0%, 5‐year survival was 58.4%, and 15‐year survival was 42.5% without significant difference between Neves levels.

**Conclusions:**

Radical nephrectomy with VTT thrombectomy and primary IVC repair is safe with high early survival and low local recurrence. Extent of IVC tumor thrombus extension is not a poor prognostic factor for early or late survival.

## 1 Introduction

Renal cell carcinoma (RCC) is a heterogeneous group of cancers arising from the renal tubular epithelium and accounts for greater than 90% of cancers of the kidney and renal pelvis [1]. In the United States, RCC is the ninth most common neoplasm with an increasing incidence since the 20th century [2]. A unique aspect of RCC is its capacity for local invasion as well as progression via venous tumor thrombus (VTT) to the renal vein, inferior vena cava (IVC), or right atrium [3]. Depending on the series, the prevalence of VTT associated with RCC is 4%−36% [3, 4, 5, 6]. Left untreated, the natural history of RCC with concomitant VTT remains poor with high disease‐specific mortality and a median life expectancy of 5 months [7]. Thus, the contemporary management of RCC with concomitant VTT continues to include radical nephrectomy and VTT thrombectomy in appropriate surgical candidates [3]. While the operative strategy has grown to include laparoscopic and robotic approaches, these remain limited to specific centers and is not appropriate for all VTT [8, 9]. The mainstay operative approach remains open surgery and is benefited by a cross‐disciplinary approach, particularly when dealing with high‐level VTT [10, 11].

The extent of VTT is considered in the American Joint Committee on Cancer (AJCC) TNM staging system and T3a, T3b, and T3c reflect renal vein, infradiaphragmatic IVC, and supradiaphragmatic IVC involvement, respectively. Additional classification schemes have arisen in an effort to further stratify and characterize the level of VTT. Of these, the most commonly utilized remains the Mayo Clinic or eponymously named Neves classification system [12]. In the Neves classification system, level 0 corresponds to renal vein involvement only, level I includes VTT extending < 2 cm into the IVC, level II includes > 2 cm into the IVC but infrahepatic, level III includes the intrahepatic IVC but infradiaphragmatic, and level IV includes the supradiaphragmatic IVC to right atrial involvement. Case series and registry‐based studies have found mixed results on the impact of VTT extent on survival and few with extended follow‐up or specific attention to the performance of vascular reconstruction [11, 13, 14, 15, 16, 17]. Given heterogeneous reports and often limited duration of follow‐up, this study sought to evaluate the impact of Neves level on perioperative and postoperative outcomes after radical nephrectomy and VTT thrombectomy performed by a dedicated team of urologists and a single vascular surgeon with extended follow‐up.

## 2 Materials and Methods

### 2.1 Data Abstraction & Statistical Analysis

Institutional Review Board approval was obtained (STU00221351) to retrospectively review all patients that had undergone radical nephrectomy for RCC with associated VTT between 2000 and 2024. Operations performed by a single vascular surgeon (M.K.E.) were identified and included for ultimate analysis. Sixty‐four patients met inclusion criteria and patient data, cancer characteristics, and outcome measures were abstracted from the electronic medical record. Patient demographics, comorbidities, and preoperative medications were obtained from outpatient and inpatient records. Neves level was identified from preoperative records and confirmed on preoperative CT or MRI. Pathologic reports were reviewed for determination of staging, histologic grade, tumor volume, and resection margin. Given the evolving classification systems across the timespan of the study, both the International Society of Urology Pathology (ISUP) and the Fuhrman grading scheme were utilized; the ISUP grading system was preferentially utilized if both grades were present. Operative details were abstracted from operative reports. Postoperative details were collected from inpatient and outpatient records. IVC patency and local tumor bed recurrence were determined from postoperative records and evaluation of postoperative CT or MRI. Patients were dichotomized by Neves level for analysis to compare infrahepatic IVC involvement (Neves 0−II) and suprahepatic IVC involvement (Neves III−IV).

Data were analyzed with Prism 10.0 (GraphPad Software, San Diego, CA) and results are presented as means with standard deviation when able. An unpaired two‐tailed *t‐*test was used for analysis of continuous variables with two groups. Chi‐squared or Fischer's exact test was used for the comparison of categorical variables. Kaplan−Meier method was utilized to construct survival curves. Mantel−Cox statistical test was utilized for analysis. A *p* value of < 0.05 was considered statistically significant.

### 2.2 Description of Operative Technique

#### 2.2.1 Neves 0−II

The abdomen is first entered by the urologic surgical team via a transabdominal, thoracoabdominal, or chevron incision. Standard techniques are employed to mobilize the colon and small intestine for adequate exposure of the infrahepatic IVC. The renal hilum is identified, and the renal artery and any accessory renal arteries are divided with a vascular load stapler. The kidney is fully mobilized, and the vascular surgical team ligates and divides all posterior lumbar venous tributaries. The IVC and major venous tributaries are controlled with vessel loops. At this point, the kidney is only attached via the stalk of the renal vein. We then turn our attention to the management of the VTT.

Control of the suprarenal and infrarenal IVC as well as the contralateral renal vein is obtained. A lateral venotomy is made at the confluence of the renal vein and IVC. In cases with VTT extension into the IVC, the VTT is milked en bloc from the venotomy and removed with the renal vein. Grossly negative margins are obtained and the IVC is thoroughly flushed. The IVC is inspected and primarily closed with 3‐0 or 4‐0 running monofilament polypropylene suture (Prolene, Ethicon, Bridgewater, NJ) resulting in no clinically significant narrowing.

#### 2.2.2 Neves III−IV

**Figure 1:**
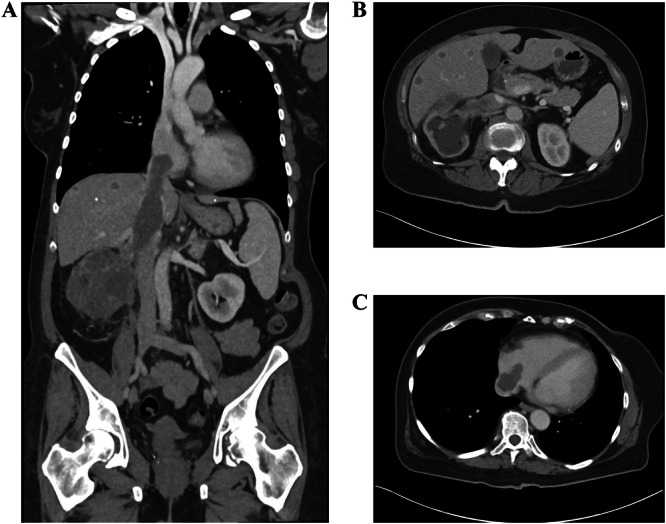
Computed tomography of a patient with renal cell carcinoma and Neves IV venous tumor thrombus. (A) Coronal view. (B) Axial view at level of renal vein. (C) Axial view at level of right atrium.

When cardiopulmonary bypass is indicated, such as for intra‐atrial VTT (Figure 1), a median sternotomy is first performed by the cardiac surgical team. The abdomen is then entered as described above for Neves 0−II. Control of the supradiaphragmatic or retrohepatic IVC is accomplished with the assistance of the vascular surgical team as well as the transplant surgical team if extensive liver mobilization is warranted. The infrahepatic IVC is controlled as described above. After mobilization of the kidney, VTT thrombectomy is then performed.

If atrial extension is identified, the VTT is milked inferior to the cavoatrial junction and supradiaphragmatic IVC control is obtained or if necessary the patient is placed on cardiopulmonary bypass and the atrium opened to remove this component of the tumor thrombus down to the infradiphragmatic location. Supradiaphragmatic VTT is, in turn, milked to the level of the hepatic veins and the suprahepatic IVC is controlled. A venotomy is extended from the level of the renal vein to the hepatic vein to remove the VTT en bloc and the clamp moved below the hepatic veins. After thrombectomy, the IVC is thoroughly flushed and primarily repaired with running 3‐0 or 4‐0 monofilament polypropylene suture resulting in no clinically significant narrowing.

## 3 Results

### 3.1 Patient Demographics and Characteristics

In total, 64 patients were included in our study, of which 34 were Neves 0−II and 30 Neves III−IV. A summary of patient characteristics is provided in Table 1. Overall, the average age was 61.5 ± 10.4 years of age, 62.5% (*n* = 40) were male, and 73.4% (*n* = 47) were White. Frequent comorbid conditions included hypertension (60.9%, *n* = 39), hyperlipidemia (37.5%, *n* = 24), and diabetes (31.3%, *n* = 20); coronary artery disease was less frequent (15.6%, *n* = 10). The majority of patients were nonsmokers (51.6%, *n* = 33) or former smokers (40.6%, *n* = 26) with very few current smokers (7.8%, *n* = 5). Few patients were prescribed preoperative antiplatelet therapy (12.5%, *n* = 8) or anticoagulation (17.2%, *n* = 11). Preoperatively identified pulmonary embolism (PE) was relatively uncommon (12.5%, *n* = 8). When comparing Neves 0−II and Neves III−IV patients, there we no significant differences between the two cohorts (Table 1).

**Table 1:** Patient characteristics: Overall and Neves 0−II versus III−IV.

	Overall (*N* = 64)	Neves 0−II (*n* = 34)	Neves III−IV (*n* = 30)	*p* value
Age (years)	61.5 ± 10.4	61.5 ± 12.2	61.4 ± 7.9	0.98
Sex				0.20
Male	62.5% (40)	70.6% (24)	53.3% (16)	
Female	37.5% (24)	29.4% (10)	46.7% (14)	
Race				0.59
White	73.4% (47)	79.4% (27)	66.7% (20)	
Black	9.4% (6)	8.8% (3)	10.0% (3)	
Hispanic	7.8% (5)	5.9% (2)	10.0% (3)	
Asian	3.1% (2)	0% (0)	6.7% (2)	
Other	1.6% (1)	2.9% (1)	0% (0)	
Declined	4.7% (3)	2.9% (1)	6.7% (2)	
Coronary artery disease	15.6% (10)	17.6% (6)	13.3% (4)	0.74
Hypertension	60.9% (39)	61.8% (21)	60.0% (18)	> 0.99
Hyperlipidemia	37.5% (24)	32.4% (11)	43.3% (13)	0.31
Diabetes mellitus	31.3% (20)	32.4% (11)	30.0% (9)	> 0.99
Smoking status				0.35
Never	51.6% (33)	44.1% (15)	60.0% (18)	
Former	40.6% (26)	44.1% (15)	36.7% (11)	
Current	7.8% (5)	11.8% (4)	3.3% (1)	
Antiplatelet therapy				
Preoperative	12.5% (8)	14.7% (5)	10.0% (3)	0.71
Postoperative	57.8% (37)	32.4% (11)	53.3% (16)	0.13
Anticoagulant therapy				
Preoperative	17.2% (11)	11.8% (4)	30.0% (9)	0.12
Postoperative	14.1% (20)	26.5% (9)	36.7% (11)	0.43
Preoperative pulmonary embolism				> 0.99
Yes	12.5% (8)	11.8% (4)	13.3% (4)	
No	87.5% (56)	88.2% (30)	86.7% (26)	

### 3.2 Tumor and Cancer Characteristics

Tumor and cancer characteristics were abstracted from the pathologic reports and the electronic medical record. A summary of tumor and cancer characteristics is provided in Table 2. The majority of RCCs involved the right kidney (71.9%, *n* = 46). Most patients presented with stage T3 cancer (T3a: 20.3%, *n* = 13; T3b 45.3%, *n* = 29; T3c 25.0%, *n* = 16) and negative nodal involvement was common (N0: 64.1%, *n* = 41). Preoperative metastases were frequent albeit not the majority (40.6%, *n* = 26). Overall, the vast majority of patients presented with Stage III (57.8%, *n* = 37) or Stage IV (39.1%, *n* = 25) RCC. Both the ISUP and Furhman grading systems were employed during the study period. Histologic grade was utilized using a composite of the two scoring systems with a preference for ISUP grade if both available. Most patient tumors demonstrated Grade 3 (43.8%, *n* = 28) or Grade 4 (46.9%, *n* = 30) ISUP/Furhman grade. The majority of RCCs were clear cell type (87.5%, *n* = 56) and the average tumor volume was 823.6 ± 828.7 cm^3^. A few patients received preoperative chemotherapy or targeted therapy (12.5%, *n* = 8). Resection margin was frequently R0 (39.1%, *n* = 25) and most commonly R1 (59.4%, *n* = 38). There was a single R2 resection in a Neves III patient in which full liver mobilization was precluded by the presence of a ventricular assist device driveline and subsequent early termination of the surgery after radical nephrectomy due to patient instability. When comparing Neves 0−II and Neves III−IV patient tumor and cancer characteristics, there were no significant differences between the two cohorts (Table 2).

**Table 2:** Tumor and cancer characteristics: Overall and Neves 0−II versus III−IV.

	Overall (*N* = 64)	Neves 0−II (*n* = 34)	Neves III−IV (*n* = 30)	*p* value
Tumor laterality				0.27
Left	28.1% (18)	35.3% (12)	20.0% (6)	
Right	71.9% (46)	64.7% (22)	80.0% (24)	
T stage				0.98
T3a	20.3% (13)	20.6% (7)	20.0% (6)	
T3b	45.3% (29)	47.1% (16)	43.3% (13)	
T3c	25.0% (16)	23.5% (8)	26.7% (8)	
T4	6.3% (4)	5.9% (2)	6.7% (2)	
Lymph node status				0.52
N0	64.1% (41)	70.6% (24)	56.7% (17)	
N1	25.0% (16)	20.6% (7)	30.0% (9)	
Nx	10.9% (7)	8.9% (3)	13.3% (4)	
Preoperative metastases				0.80
Yes	40.6% (26)	38.2% (13)	43.3% (13)	
No	59.4% (38)	61.8% (21)	56.7% (17)	
Cancer stage				0.12
Stage I	1.6% (1)	2.9% (1)	0% (0)	
Stage II	1.6% (1)	2.9% (1)	0% (0)	
Stage III	57.8% (37)	47.1% (16)	70.0% (21)	
Stage IV	39.1% (25)	47.1% (16)	30.0% (9)	
ISUP/Fuhrman grade				0.72
Grade 1	0% (0)	0% (0)	0% (0)	
Grade 2	6.3% (4)	8.8% (3)	3.3% (1)	
Grade 3	43.8% (28)	44.1% (15)	43.3% (13)	
Grade 4	46.9% (30)	44.1% (15)	50.0% (15)	
Histologic type				0.71
Clear cell	87.5% (56)	82.4% (28)	93.3% (28)	
Papillary	3.1% (2)	5.9% (2)	3.3% (1)	
Chromophobe	3.1% (2)	5.9% (2)	0% (0)	
Unclassified	3.1% (2)	5.9% (2)	3.3% (1)	
Tumor volume (cm^3^)	823.6 ± 828.7	902.5 ± 952.3	731.0 ± 66	0.42
Preoperative chemotherapy or targeted therapy				> 0.99
Yes	12.5% (8)	11.8% (4)	13.3% (4)	
No	87.5% (56)	88.2% (30)	86.7% (26)	
Resection margin				0.20
R0	39.1% (25)	47.1% (16)	30.0% (9)	
R1	59.4% (38)	52.9% (18)	66.7 (20)	
R2	1.6% (1)	0% (0)	3.3% (1)	

### 3.3 Operative Characteristics

Operative details were abstracted from operative records. A summary of operative characteristics is provided in Table 3. Average operative duration was 344.0 ± 134.6 min and there was no significant difference between Neves 0−II and Neves III−IV patients (322.1 ± 133.5 vs. 369.5 ± 133.6 min, *p* = 0.17). There was only a single instance of cardiopulmonary bypass usage for a Neves II patient in which there was an intraoperative VTT embolism with subsequent hypotension. Otherwise, cardiopulmonary bypass was exclusively utilized in Neves III−IV patients (16.7%, *n* = 5), however, this difference was not statistically significant (*p* = 0.10). Significant blood loss (> 2 L) was common (45.3%, *n* = 29) and significantly more frequent in Neves III−IV patients (60.0%, *n* = 18) than Neves 0−II patients (32.4%, *n* = 11, *p* = 0.02).

**Table 3:** Operative characteristics: Overall and Neves 0−II versus III−IV.

	Overall (*N* = 64)	Neves 0−II (*n* = 34)	Neves III−IV (*n* = 30)	*p* value
Operative duration (min)	344.0 ± 134.6	322.1 ± 133.5	369.5 ± 133.6	0.17
Cardiopulmonary bypass				0.10
Yes	9.4% (6)	2.9% (1)	16.7% (5)	
No	90.6% (58)	97.1% (33)	83.3% (25)	
Estimated blood loss > 2 L				0.02
Yes	45.3% (29)	32.4% (11)	60.0% (18)	
No	50.0% (32)	64.7% (22)	33.3% (10)	

### 3.4 Postoperative Characteristics

Postoperative details were abstracted from inpatient outpatient records. A summary of postoperative characteristics is provided in Table 4. Overall, the average intensive care unit (ICU) length of stay (LOS) was 2.8 ± 5.4 days and Neves 0−II patients demonstrated significantly shorter ICU LOS than Neves III−IV patients (1.4 ± 1.7 vs. 4.4 ± 7.4 days, *p* = 0.02). Similarly, overall postoperative LOS was 8.6 ± 6.4 days and Neves 0−II demonstrated significantly shorter postoperative LOS than Neves III−IV patients (6.5 ± 3.4 vs. 11.0 ± 8.0 days, *p* = 0.005). Preoperative creatinine was 1.33 ± 1.40 mg/dL with a slight increase to 1.62 ± 1.0 mg/dL on postoperative Day 1 and 1.61 ± 1.2 mg/dL on postoperative Day 3. There was no significant difference between Neves 0−II and Neves III−IV patients.

**Table 4:** Postoperative characteristics: Overall and Neves 0−II versus III−IV.

	Overall (*N* = 64)	Neves 0−II (*n* = 34)	Neves III−IV (*n* = 30)	*p* value
ICU length of stay (days)	2.8 ± 5.4	1.4 ± 1.7	4.4 ± 7.4	0.02
Postoperative length of stay (days)	8.6 ± 6.4	6.5 ± 3.4	11.0 ± 8.0	0.005
Perioperative creatinine				
Preoperative	1.33 ± 1.40	1.1 ± 0.3	1.6 ± 2.0	0.22
Postoperative Day 1	1.62 ± 1.0	1.6 ± 0.6	1.7 ± 1.4	0.79
Postoperative Day 3	1.61 ± 1.2	1.6 ± 0.9	1.6 ± 1.5	0.93
30‐day morbidity				0.10
Yes	17.2% (11)	8.8% (3)	26.7% (8)	
No	83.8% (53)	91.2% (31)	73.3% (22)	
30‐day postoperative pulmonary embolism				> 0.99
Yes	6.3% (4)	5.9% (2)	6.7% (2)	
No	93.7% (60)	94.1% (32)	93.3% (28)	
30‐day mortality				> 0.99
Yes	1.6% (1)	2.9% (1)	0% (0)	
No	98.4% (63)	97.1% (33)	100% (30)	
Postoperative IVC patency				0.22
Yes	96.9% (62)	100% (34)	93.3% (28)	
No	3.1% (2)	0% (0)	6.7% (2)	
Local tumor bed recurrence				0.66
Yes	7.8% (5)	5.9% (2)	10.0% (3)	
No	92.2% (59)	94.1% (32)	90.0% (27)	

Postoperative morbidity, including myocardial infraction, respiratory failure, renal failure, new PE, and reoperation for bleeding, was evaluated. Thirty‐day morbidity was 17.2% (*n* = 11) and more frequent among the Neves III−IV patients (26.7%, *n* = 8) but this was not statistically significant. New postoperative PE was uncommon (6.3%, *n* = 4) without significant difference between Neves 0−II and Neves III−IV patients. Thirty‐day mortality was also rare and limited to only the previously mentioned patient with intraoperative VTT embolism requiring cardiopulmonary bypass (1.6%, *n* = 1). Postoperative IVC patency was assessed via clinical and radiographic findings. Patency was excellent (96.9%, *n* = 62) and only two instances of IVC occlusion were identified in Neves III−IV patients, though this was not significant. Local tumor bed recurrence was also rare (7.8%, *n* = 5) without a significant difference between Neves 0−II and Neves III−IV patients. Notably, additional evaluation of recurrence by resection margin identified no significant differences (R0: 8.0%, *n* = 2 vs. R1: 7.9%, *n* = 3 vs. R2: 100.0%, *n* = 1, *p* = 0.12).

### 3.5 Long‐Term Follow‐Up and Survival

**Figure 2:**
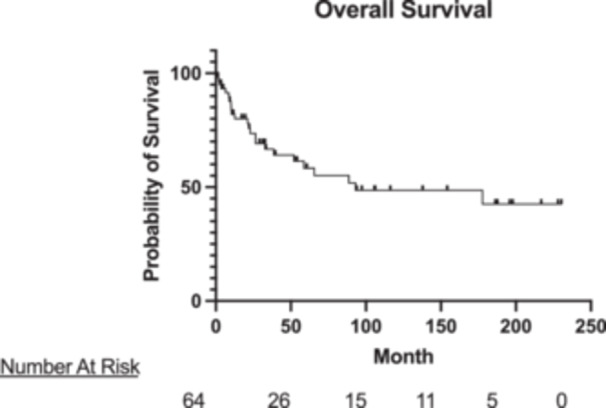
Overall survival of patients undergoing radical nephrectomy and thrombectomy for renal cell carcinoma with venous tumor thrombus.

**Figure 3:**
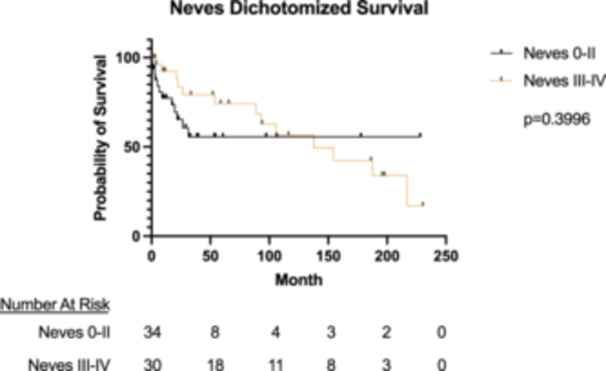
Dichotomized survival of Neves 0−II versus Neves III−IV patients undergoing radical nephrectomy and thrombectomy for renal cell carcinoma with venous tumor thrombus.

The mean follow‐up of the overall cohort was 56.1 ± 66.7 months. The median follow‐up was 26.4 months. Overall, 1‐year survival was 82.0%, 5‐year survival was 58.4%, and 15‐year survival was 42.5% (Figure 2). When evaluating Neves 0−II alone, 1‐year survival was 77.4%, 5‐year survival was 55.7%, and 15‐year survival was 55.7%. When evaluating Neves III−IV alone, 1‐year survival was 92.4%, 5‐year survival was 74.3%, and 15‐year survival was 42.4%. There was no significant difference between groups on survival analysis (*p *= 0.40) (Figure 3).

## 4 Discussion

Despite a moderate number of patients, our study provides unique insights into the management of RCC with VTT. Importantly, this series consists only of patients undergoing radical nephrectomy with VTT thrombectomy performed by a team of a few dedicated urologists and a singular vascular surgeon. Average operative duration for the entire cohort was under 6 h and not significantly increased for Neves III−IV patients in which transplant and cardiac surgical teams were also frequently involved. Additionally, there was only a single early postoperative death. Our strategy further highlights the benefits of a dedicated cross‐disciplinary and team‐based approach to this challenging surgical condition [10].

Given the divergent operative strategies for venous thrombectomy, as well as the limited patient cohorts, we elected to dichotomize the total cohort into Neves 0−II and Neves III−IV groups for analysis. We found that the extent of VTT did not correlate with patient demographics or characteristics. Nor did we find VTT extent to correlate with other tumor and cancer characteristics, including resection margin. However, we did find that Neves III−IV resection was associated with greater estimated blood loss and both ICU and postoperative LOS versus Neves 0−II patients. Lastly, the extent of VTT demonstrated no correlation with overall early or late survival as well as local tumor recurrence.

We found primary repair of the IVC, even for high level VTT, demonstrated excellent patency on long‐term follow‐up, consistent with other series [18]. Notably, no patients required a patch venoplasty or interposition venous bypass. We find a lateral venorrhaphy is adequate for complete extraction of the VTT and can be closed without significant caval stenosis. Nevertheless, the two instances of postoperative IVC occlusion were identified in a Neves III and a Neves IV patient. This may reflect the sequelae of greater caval exposure and instrumentation. Prior large series have described the need for patch venoplasty, particularly in Neves IV patients, and we similarly advocate for the use of a patch when concerned for significant narrowing [19, 20]. The need for caval replacement has been described, though the optimal conduit is not completely known [20]. In these rare instances, we suggest the use of a ringed PTFE graft, which appears to offer excellent patency when employed for caval reconstruction for other oncologic reconstructions, such as primary leiomyosarcoma of the IVC [21, 22]. Larger series and prospective studies are still necessary to determine if PTFE confers a similar advantage in this clinical context.

Local tumor recurrence was uncommon in our series and did not correlate with Neves level or resection margin. While our study is likely underpowered to detect a significant difference, other reports have found a positive resection margin to predict local recurrence [23]. However, the clinical impact of local recurrence may be limited, as the incidence of local recurrence in the absence of systemic recurrence is potentially quite rare [24]. Invasion of RCC into the vein wall may instead reflect an aggressive tumor biology with significant risk for systemic recurrence irrespective of vein margin. Interestingly, when evaluating the performance of partial nephrectomy for renal tumors, a positive margin does not necessarily portend an elevated risk of local recurrence [25]. In our practice, while an R0 resection is ideal, we prioritize the expeditious removal of VTT with maximal preservation of caval wall and frequently accept an R1 resection. This and other factors have helped obviate the need for patch venoplasty or caval reconstruction in our experience.

Despite our prolonged period of follow‐up, we found that VTT extent did not predict overall survival. This contrasts with two of the largest series of RCC with VTT that detected significant survival differences between VTT involving the renal vein alone and more superior VTT extent [26, 27]. However, several other reports, including pooled multicenter and large single retrospective studies have indicated VTT extent bears little weight on survival [14, 15, 17]. Instead, other factors such as lymph node status, preoperative metastasis status, histologic grade, or perirenal fat invasion may better predict overall survival [15, 28].

Our study is not without limitations and includes those inherent to a single‐center retrospective study. Due to the extent of the study period and shifting clinical practices, some of the patients in this series underwent neoadjuvant chemotherapy or targeted therapy. The impact of these treatments on subsequent operative and postoperative outcomes is difficult to predict given the overall paucity of their effects on VTT. Similarly, though not statistically significant between Neves 0−II and III−IV cohorts, a significant portion of our total cohort had preoperative metastases, which has potential to impact overall survival. Lastly, given limitations of routine pathologic reports, we are unable to comment on specific VTT features, such as friability, which may play a role in stratifying VTT [29].

## 5 Conclusions

In summary, we describe a series of patients with advanced RCC and VTT, including a substantial proportion of Neves III−IV patients, undergoing radical nephrectomy and VTT thrombectomy. Our findings suggest VTT thrombectomy with primary IVC repair is safe with high early and late survival rates as well as low recurrence. Additionally, the extent of venous tumor extension was not a poor prognostic factor even though suprahepatic extension was associated with a more complex operation, increased EBL, and longer LOS. Continued investigation into the prognostic value of VTT extent and strategies for VTT thrombectomy and caval repair remain important areas of investigation.

## Conflicts of Interest

M.K.E., disclosures include paid consultants for W.L. Gore and Silkroad Medical. The other authors declare no conflicts of interest.

## Synopsis

Venous tumor thrombus (VTT) extent is associated with greater blood loss and length of stay after radical nephrectomy and VTT thrombectomy for advanced renal cell carcinoma. Inferior vena cava tumor thrombus extent is not a poor prognostic factor for early or late survival. Radical nephrectomy with VTT thrombectomy and primary IVC repair is safe with low recurrence rates.

## Data Availability

The data that support the findings of this study are available from the corresponding author upon reasonable request.
